# Life‐Threatening Necrotizing Pneumonia in a Pediatric Patient: A Complicated Case From Palestine

**DOI:** 10.1155/crpe/7410769

**Published:** 2026-05-26

**Authors:** Hani Abu-Hijleh, Nagham Kawa, Seleen Abu Alhmam, Khaled Swafta, Maysaa Alawneh, Abdulkareem Saymeh, Abdulkarim Adas

**Affiliations:** ^1^ Department of Medicine, Faculty of Medicine and Health Sciences, An-Najah National University, Nablus, State of Palestine, najah.edu; ^2^ Department of Pediatrics, An-Najah National University, Nablus, State of Palestine, najah.edu; ^3^ Department of Medicine, Faculty of Medicine and Health Sciences, Al-Quds University, Jerusalem, State of Palestine, upm.edu.my; ^4^ Department of Cardiothoracic Surgery, An-Najah National University Hospital, Nablus, State of Palestine, najah.edu

**Keywords:** acute kidney injury, hemothorax, influenza, necrotizing pneumonia, pediatric

## Abstract

Necrotizing pneumonia (NP) is a severe and life‐threatening complication of lung infection in children. While rare, it can lead to catastrophic complications such as hemothorax, where blood fills the pleural cavity. We present the case of a previously healthy 5‐year‐old girl who initially appeared to have a gastrointestinal illness. Her condition rapidly evolved into a complex medical emergency involving influenza‐associated NP, which was then complicated by a sudden, massive hemothorax. Her hospital course was further challenged by drug‐induced acute kidney injury (AKI) requiring temporary hemodialysis and the persistent mystery of negative bacterial cultures. This case highlights the aggressive potential of NP, the critical need for vigilance when clinical signs worsen abruptly, and the essential role of timely surgical intervention. Following an urgent thoracotomy and lung resection, the child made a full recovery, underscoring how a multidisciplinary approach can be life‐saving in these extreme scenarios.

## 1. Introduction

Community‐acquired pneumonia is a common illness in children, and the majority of cases resolve with appropriate antibiotic therapy. However, a small proportion of cases progress to complicated pneumonia, which includes parapneumonic effusion, empyema, lung abscess, and necrotizing pneumonia (NP) [1]. NP represents one of the most severe forms, characterized by liquefactive necrosis and cavitation of the lung parenchyma, often leading to a prolonged and complicated clinical course [2]. Compared to uncomplicated pneumonia, NP is associated with prolonged fever, persistently elevated inflammatory markers, and a significantly extended hospital stay [3].

The global incidence of NP in children is rising, though it remains a rare entity. It is most commonly linked to specific bacterial pathogens, particularly *Streptococcus pneumoniae* (especially Serotypes 3 and 19A) and *Staphylococcus aureus*. The pathophysiology involves intense inflammation, thrombosis of small pulmonary vessels, and subsequent necrosis of lung tissue. The presence of a viral co‐infection, such as influenza, can exacerbate disease severity by disrupting the respiratory epithelium and impairing local immune defenses, thereby creating a fertile environment for bacterial superinfection and enhancing the destructive inflammatory response [4, 5].

Among the rare but life‐threatening complications of NP is hemothorax, the accumulation of blood within the pleural space. In the context of pediatric pneumonia, hemothorax typically results from the erosion of a pulmonary or pleural vessel secondary to the extensive parenchymal necrosis [6]. This complication is exceptionally uncommon because the necrotic process usually involves smaller, distal vessels, and the resulting inflammation can sometimes lead to thrombosis, which may paradoxically protect against hemorrhage. When a larger vessel is eroded, however, the clinical presentation is abrupt, often with hemoptysis, acute respiratory compromise, and hemodynamic instability, necessitating emergent surgical intervention [7].

Here, we report the complicated clinical course of a young girl with influenza‐associated NP who developed a spontaneous, life‐threatening hemothorax. The clinical trajectory was marked by diagnostic challenges, including persistently negative microbiological cultures, and significant management complications, such as vancomycin‐induced acute kidney injury (AKI). This case aims to illustrate the rapid and severe deterioration that can occur, emphasize the importance of prompt surgical intervention, and contribute to the clinical understanding of this rare but critical complication in pediatric pneumonia.

## 2. Case Presentation

A previously healthy 5‐year‐old girl presented to our emergency department with a 1‐week history of high‐grade fever (occurring every 3‐4 h and responding poorly to antipyretics) and an intermittent cough. Her family reported no known sick contacts. In the 24 h prior to admission, she developed abdominal pain and tachypnea, prompting this presentation.

On physical examination, the patient was in significant respiratory distress, characterized by tachypnea (respiratory rate 48 breaths/min), grunting, and subcostal retractions. Her vital signs on admission were as follows: heart rate 145 beats/min, blood pressure 98/58 mmHg, and oxygen saturation 88% on room air, which improved to 94% with 2 L/min of oxygen via nasal cannula. Auscultation revealed diffuse bilateral crepitations with markedly decreased air entry in the left lower lung zone. The remainder of the examination was unremarkable. Initial laboratory investigations are summarized in Table 1.

**TABLE 1:** Laboratory investigations on admission.

Test category	Parameter	Result
Complete blood count (CBC)	WBC	7.77 × 10^9^/L
	Hemoglobin (Hb)	10.7 g/dL
	Mean corpuscular volume (MCV)	70 fL
	Platelets	218 × 10^9^/L
	Neutrophils	77%

Inflammatory markers	C‐Reactive protein (CRP)	391 mg/L
	Erythrocyte sedimentation rate (ESR)	78 mm/hr

Viral testing	Influenza A	Positive
	Influenza B	Negative
	COVID‐19 (rapid test)	Negative

Coagulation profile	Prothrombin time (PT)	14.3 s
	INR	1.16
	Partial thromboplastin time (PTT)	38 s
	Fibrinogen	981 mg/dL

Liver function tests (LFTs)	Albumin	3.31 g/dL
	Alkaline phosphatase (ALP)	137 U/L
	Total serum bilirubin (TSB)	0.47 mg/dL
	AST	30 U/L
	ALT	15.1 U/L

Renal function and electrolytes	Blood urea nitrogen (BUN)	5.88 mg/dL
	Creatinine	0.26 mg/dL
	Calcium	8.96 mg/dL
	Phosphate	2.41 mg/dL
	Magnesium	1.87 mg/dL
	Sodium	134.6 mmol/L
	Potassium	4.18 mmol/L
	Chloride	99.1 mmol/L

Other tests	Lactate dehydrogenase (LDH)	341 U/L
	Total protein	5.76 g/dL

Microbiology	Urine streptococcal antigen	Negative

**FIGURE 1:**
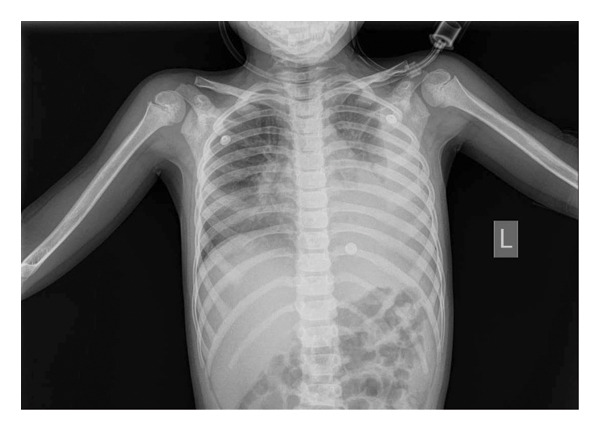
Chest X‐ray on admission demonstrating a large left‐sided pleural effusion with mediastinal shift.

**FIGURE 2:**
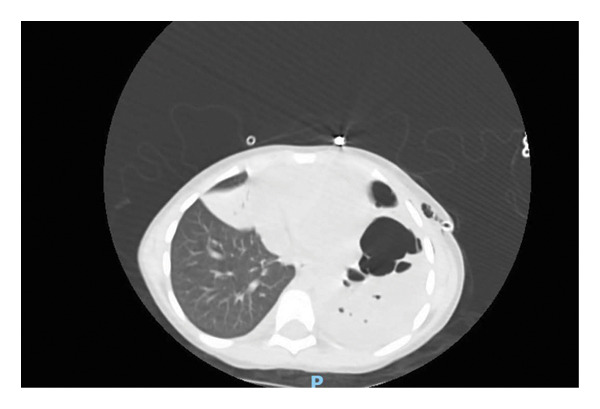
Axial contrast‐enhanced CT scan on admission showing left lower lobe consolidation with areas of hypoattenuation and internal air locules, consistent with necrotizing pneumonia. A large left pleural effusion is also present.

Chest radiography showed left lower lobe consolidation with a large pleural effusion (Figure 1). A chest CT scan with IV contrast confirmed left‐sided NP (Figure 2). The patient was admitted and started on empiric intravenous antibiotics (ceftriaxone and vancomycin). A chest tube was inserted by the surgical team, draining approximately 200 mL of turbid pleural fluid, which was consistent with an exudative effusion by Light’s criteria. Gram stain and culture of the fluid were negative. Following drainage, the patient was started on oseltamivir for confirmed Influenza A and azithromycin, while ceftriaxone and vancomycin were continued.

Despite this intervention, the patient continued to have high‐grade fevers with persistently elevated inflammatory markers. Due to the lack of clinical improvement, antibiotics were escalated on Day 4 to piperacillin–tazobactam and vancomycin. Serial blood cultures, taken every 48 h, remained negative.

On Day 7 of admission, the patient’s renal function deteriorated. A vancomycin trough level returned at 28 μg/mL (therapeutic range: 10–20 μg/mL), and serum creatinine had risen from a baseline of 0.26 mg/dL to 2.1 mg/dL, consistent with AKI. Vancomycin was discontinued, and antibiotic coverage was narrowed to meropenem. Due to worsening AKI (peak creatinine: 3.4 mg/dL; BUN: 58 mg/dL) with oliguria, a temporary dialysis catheter was inserted, and the patient underwent four sessions of hemodialysis over the subsequent week.

On Day 10 of admission, the patient developed a sudden episode of forceful coughing followed by massive hemoptysis. Moments later, a large volume of fresh blood gushed from the chest tube, amounting to approximately 650 mL, indicating a life‐threatening left‐sided hemothorax. The patient became tachycardic (HR: 170 bpm) and hypotensive (BP: 75/40 mmHg).

Resuscitation was initiated immediately, including intravenous fluids, nebulized and intravenous tranexamic acid, fresh frozen plasma (FFP), and a transfusion of 2 units of packed red blood cells. A repeat hemoglobin level dropped to 4 g/dL. Coagulation studies remained normal.

An urgent left‐sided thoracotomy was performed. Upon entering the pleural cavity, approximately 400 mL of hemothorax and an organized hematoma were evacuated. A left lower lobe segmentectomy was performed. Intraoperative findings revealed extensive necrosis and destruction of the left lower lobe, with areas of active bleeding from necrotic pleural and parenchymal surfaces. The source of hemorrhage was confirmed to be erosion of a segmental pulmonary vessel within the necrotic cavity.

**FIGURE 3:**
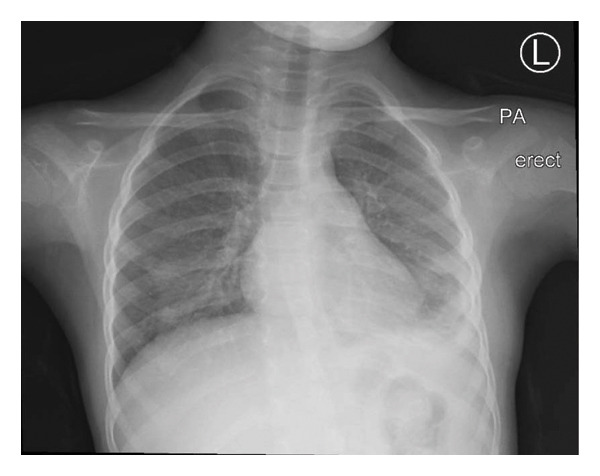
Follow‐up chest X‐ray prior to discharge showing decreased left lower zone opacity and interval resolution of the large pleural effusion, with residual left basilar opacities.

**FIGURE 4:**
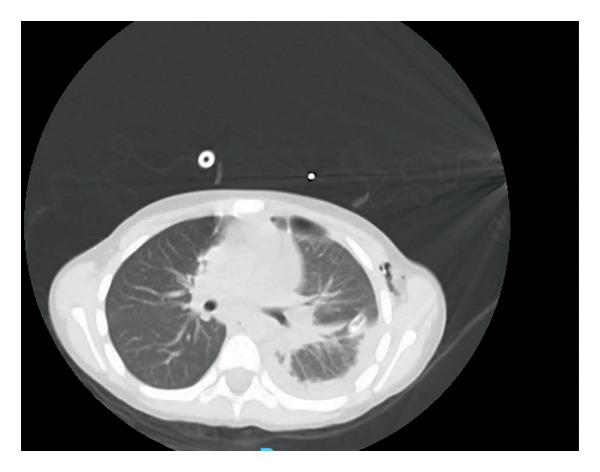
Axial noncontrast CT scan prior to discharge demonstrating interval progression of cavitation within the left lower lobe following segmentectomy, with a small loculated pneumothorax. The left pleural effusion has significantly decreased.

Postoperatively, the patient remained intubated for one day. Her fever began to subside within 48 h, and inflammatory markers trended downward. She was extubated successfully on Postoperative day 2, and the chest tube was removed on Day 5. Once she was afebrile for 48 h, antibiotics were transitioned to an oral regimen, and she was observed for any recurrence of fever. Repeat chest imaging (Figures 3 and 4) showed improvement. All follow‐up cultures remained negative, and her last CRP before discharge was 40 mg/L. She was discharged home on oral antibiotics for a total of 4 weeks, with normalizing renal function.

## 3. Discussion

This case presents a severe and rapidly progressive episode of NP in a previously healthy 5‐year‐old child, complicated by a massive, life‐threatening hemothorax. While NP itself is a recognized severe complication of pediatric pneumonia, the specific clinical trajectory and confluence of critical events in this case provide a unique contribution to the literature, highlighting extreme management challenges and reinforcing critical principles in pediatric critical care.

What makes this presentation especially distinctive is the simultaneous occurrence of three critical, interdependent conditions: (1) Influenza A‐associated NP resistant to broad‐spectrum antibiotics, (2) a catastrophic massive hemothorax resulting from parenchymal erosion, and (3) a significant iatrogenic AKI due to vancomycin toxicity, which required temporary hemodialysis. The addition of AKI introduced a complex comorbidity that complicated fluid management, drug dosing, and hemodynamic stability ahead of the emergency surgery needed for the hemothorax. Few pediatric cases in the literature describe the management of a massive NP‐associated hemothorax, and even fewer do so in the concurrent setting of medically‐induced renal failure. This narrative serves as a critical example of successful multidisciplinary management involving infectious disease, nephrology, and thoracic surgery teams.

The child’s initial complaint of abdominal pain highlights how NP can mimic surgical abdominal pathologies, a factor that can potentially delay accurate diagnosis [2]. The identification of Influenza A co‐infection is a key etiological factor. Influenza virus is known to disrupt the respiratory epithelium and impair mucociliary clearance and innate immunity, thereby predisposing patients to severe secondary bacterial infections and aggressive lung destruction [5]. This viral–bacterial synergy likely contributed to the rapid and extensive necrosis seen in our patient.

Hemothorax is an exceptionally rare complication of NP. The pathophysiology involves the erosion of a pulmonary or pleural vessel wall by the intense inflammatory and necrotic process. This is uncommon because the necrotizing process typically affects smaller, peripheral vessels, and the accompanying thrombosis may sometimes seal off vessels before catastrophic hemorrhage can occur [6]. However, when a larger, more central vessel is involved, as suspected in our case, the result is a life‐threatening surgical emergency. The failure of chest tube drainage alone to manage this complication is a critical teaching point: drainage addresses the secondary accumulation of blood in the pleural space but does not remove the source—the necrotic, bleeding lung parenchyma. Definitive control requires resection of the involved tissue, as was successfully performed here.

The literature on NP‐associated hemothorax is sparse. A targeted PubMed search using keywords (“necrotizing pneumonia” AND “hemothorax”) reveals important patterns. Table 2 summarizes and compares the management strategies in relevant reported cases.

**TABLE 2:** Comparison of management strategies in necrotizing pneumonia and lung abscess complications.

Study (author, year)	Patient age	Condition	Complication	Management	Outcome	Key takeaway
Rigby et al. (2012) [8]	8y (pediatric)	NP + vasculitis	Tension pneumothorax and hemothorax	Medical: Factor VIIa + drainage	Survived	Hemorrhage controlled with medical therapy in a patient on ECMO
Jung et al. (2021) [9]	63y (adult)	COVID‐19 NP	Massive hemothorax	Surgical: emergency lobectomy	Survived	Emergent surgical resection is required for massive, active hemorrhage
Ghabally et al. (2024) [10]	52y (adult)	COVID‐19 pneumonia	Abrupt massive hemothorax	Drainage only: thoracostomy	Died	Drainage alone is insufficient for uncontrolled parenchymal bleeding
Current case	5 y (pediatric)	Influenza A + NP	Massive hemothorax + AKI	Surgical: segmentectomy	Survived	Surgical resection is definitive; it highlights the need for multidisciplinary care

The literature comparison underscores three key points relevant to our case: (1) The management choice is dictated by the acuity of hemorrhage, with ongoing massive bleeding typically requiring urgent surgical resection for survival; (2) drainage alone can be insufficient if the necrotic, bleeding parenchymal nidus is not removed, as tragically illustrated by Ghabally et al. [10]; and (3) underlying etiology matters—young children with viral–bacterial co‐infection may progress rapidly to catastrophic complications despite broad‐spectrum antibiotics. Our patient’s postoperative course strongly supports an operative approach when structural bleeding from necrotic tissue is present.

The development of vancomycin‐induced nephrotoxicity, severe enough to require hemodialysis, is a serious and well‐documented risk of aggressive dosing, particularly in critically ill patients [11]. In this child, it represented a major iatrogenic comorbidity, emphasizing the absolute necessity for vigilant therapeutic drug monitoring in pediatric sepsis. This complication also forced a difficult balance between providing adequate antibiotic coverage and managing fluid status and electrolyte balance in a patient at risk for hemorrhagic shock.

Reflecting on lessons learned, one might ask if earlier surgical consultation was warranted. The patient’s persistent sepsis and lack of response to broad‐spectrum antibiotics, even before the hemothorax, could be considered an indication for surgical debridement of the necrotic focus. While surgical intervention for NP without a complication such as hemothorax remains controversial, this case suggests that a lower threshold for involving a thoracic surgeon in cases of medically refractory NP may be beneficial.

## 4. Conclusion

This case highlights the severity of influenza‐associated NP in children and the critical need to anticipate rare, life‐threatening complications such as massive hemothorax. It underscores the dual challenges of managing aggressive infection—requiring prompt surgical intervention for structural complications—while vigilantly avoiding iatrogenic harm from nephrotoxic antibiotics. Successful outcomes depend on early recognition, multidisciplinary coordination, and a readiness to escalate to definitive surgical management when medical therapy fails [12].

## Funding

No specific grant from funding agencies was received for this work.

## Ethics Statement

Our institution does not require ethical approval for reporting individual case reports or case series.

## Consent

Written informed consent was obtained from the patient’s parent/guardian for the publication of this case report and any accompanying images.

## Conflicts of Interest

The authors declare no conflicts of interest.

## Data Availability

The data that support the findings of this study are available from the corresponding author upon reasonable request.
